# *Chitinophaga terrae* Bacteremia in Human

**DOI:** 10.3201/eid1507.090124

**Published:** 2009-07

**Authors:** Lise Crémet, Pascale Bemer, Olivier Zambon, Alain Reynaud, Nathalie Caroff, Stéphane Corvec

**Affiliations:** Nantes University Hospital, Nantes, France

**Keywords:** Chitinophaga, bacteremia, opportunistic pathogens, letter

**To the Editor:** The genus *Chitinophaga,* first described by Sangkhobol and Skerman in 1981, belongs to the phylum Bacteroidetes (formerly the *Cytophaga*-*Flexibacter*-*Bacteroides* group), which includes filamentous, chitinolytic, gliding bacteria that transform into spherical bodies upon aging (*1*). This genus contains 10 environmental species that demonstrate similarities in 16S rDNA sequence and in phenotypic and chemotaxonomic data (menaquinone, fatty acids, hydroxy fatty acid, and polyamine) (*2*–*5*). *Chitinophaga terrae*, originally isolated from soil in South Korea, was first described in 2007 (*3*,*4*). Here we report a case of bacteremia due to *C. terrae* in a severely immunosuppressed woman.

On July 31, 2008, a 51-year-old woman was admitted to the emergency department at Nantes University Hospital in Nantes, France, because of a slowly growing left cheek mass associated with weight loss and change of general state. Physical examination showed several cutaneous infiltrated nodules, bilateral axillary adenopathies, and hepatosplenomegaly. Deteriorating renal function led to intermittent hemodialysis. Histopathology of skin and renal biopsies revealed a diffuse, high–grade, large B-cell lymphoma with cutaneous localization. Systemic CHOP (cyclophosphamide, doxorubicin, vincristine, prednisone) chemotherapy and methotrexate intrathecal chemotherapy were begun August 9. She developed bone marrow aplasia 3 days later, shortly followed by the onset of pyrexia. *Corynebacterium pseudotuberculosis* was isolated from 3 blood samples, 2 drawn using the central venous catheter (CVC) and 1 from peripheral blood. Consecutively, serotype O1 *Pseudomonas aeruginosa* strain was isolated from cultures of urine and fecal specimens. The empirical antimicrobial drug treatment started with piperacillin-tazobactam, ciprofloxacin, and teicoplanin on August 16 and was replaced by imipenem, ciprofloxacin, and teicoplanin on August 21. On August 26, the patient was admitted to the medical intensive care unit (MICU) after indications of toxic epidermal necrolysis. Blood analysis showed pancytopenia with thrombocytopenia (26 × 10^9^/L), anemia (hemoglobin 8.7 g/dL), and profound leukopenia (0.01 × 10^9^/L). Antimicrobial drug treatment was changed to an association of noncytotoxic drugs, i.e., aztreonam, amikacin, and teicoplanin. Despite this broad-spectrum antimicrobial therapy, 4 aerobic blood cultures (1 drawn August 29, 2 on September 2, and 1 on September 3), 2 drawn using the CVC and 2 from a peripheral site, yielded gram-negative bacilli (our laboratory reference no. NTS8639) after 2 days’ incubation. The CVC was removed September 3 and sent to the laboratory for culture. The catheter tip was immersed in 2 mL of brain heart infusion agar, and semiquantitative culture was performed on the blood agar plate using 100 µL of the solution. The culture remained negative.

On September 2, treatment was changed to imipenem, trimethoprim-sulfamethoxazole, and teicoplanin. Additionally, diagnosis of invasive pulmonary aspergillosis led to changing caspofungin prophylaxis to voriconazole. Trimethoprim-sulfamethoxazole treatment was stopped September 9, and imipenem was stopped September 25, a week after bone marrow recovery. The patient was discharged from MICU on October 7.

Yellow-pigmented colonies grew on bromocresol purple lactose agar plate after 48 hours of incubation at 37°C and appeared as thin gram-negative bacilli after gram-staining was performed. The nonfermenting, nonmotile, oxidase-positive bacterium could grow at various pH values (pH 6.0, 7.3, and 8.0) and at different temperatures (30, 37, and 40°C). The semi-automatic Api 20NE gallery (bioMérieux, Marcy l’Etoile, France) identified the strain as *Sphingomonas paucimobilis*, whereas the ID-GNB card of the VITEK 2 system (bioMérieux) identified the bacterium as *Sphingobacterium thalpophilum*. The 16S rDNA amplification and sequencing were performed with universal primers 27f and 1378r as previously described (*6*). The 1366-bp sequence matched that of *C. terrae* with 100% similarity, according to BIBI (Bioinformatic Bacteria Identification, http://umr5558-sud-str1.univ-lyon1.fr/lebibi/lebibi.cgi) or BLAST (www.ncbi.nlm.nih.gov) analysis. Phylogenetic analysis with either the neighbor-joining or maximum-parsimony algorithm embedded the NTS8639 strain to the genus *Chitinophaga* and the species *C. terrae* (Figure). The biochemical characteristics of the bacterium corresponded to those previously described for *C. terrae* by Kim and Jung (*3*). The strain reduced nitrate to nitrite, produced N-acetyl-β-glucosamidase, phosphatase, α- and β-galactosidases, α- and β-glucosidases, and assimilated L-arabinose, L-fucose, D-glucose, maltose, D-mannose, D-melibiose, L-rhamnose, sucrose and salicin with Api 20NE, Api ID32GN (bioMérieux), and ID-GNB biochemical galleries. Unlike *S. paucimobilis*, the strain was positive for nitrate reductase and L-rhamnose. Also, negative reaction for urease and L-fucose assimilation differentiated the strain from *S. thalpophilum*. At the species level, the bacterium grew well at 37°C, unlike *Chitinophaga arvensicola*, and showed positive oxidase reaction and nitrate reduction, unlike *Chitinophaga ginsengisegetis* (*2*,*5*).

**Figure:**
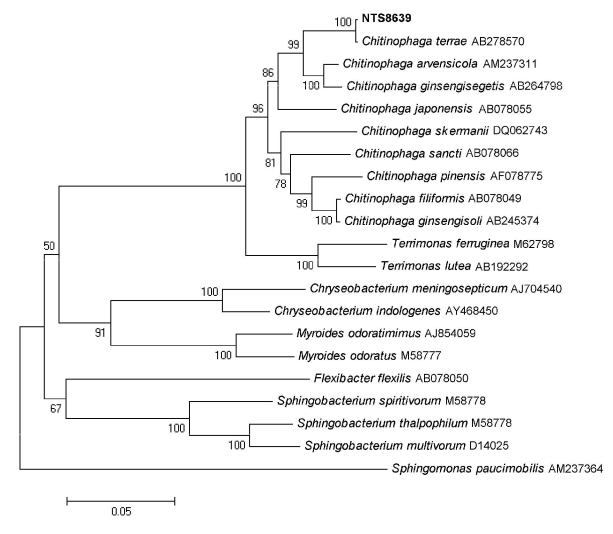
Neighbor-joining (NJ) tree showing the phylogenetic placement of strain NTS8639 (in **boldface**) among members of the *Chitinophaga terrae* species. Twenty-one 16S rRNA gene sequences selected from the GenBank database were aligned with that of strain NTS8639 by using MEGA4 (www.megasoftware.net). Accession numbers are indicated after the species name. The evolutionary history was inferred using the NJ method. The figure shows the optimal tree; the sum of the branch lengths = 1.12829943. The percentage of replicate trees in which the associated taxa clustered together in the bootstrap test (1,000 replicates) are shown next to the branches. The tree is drawn to scale, with branch lengths in the same units as those of the evolutionary distances used to infer the phylogenetic tree. The evolutionary distances were computed using the Kimura 2–parameter method and are in the units of the number of base substitutions per site. The final dataset contains 1,304 positions. Phylogenetic analyses were conducted in MEGA4. NJ and parsimony trees were globally congruent with the distance tree and confirmed the placement of the strain NTS8639 in the *C. terrae* species. Scale bar indicates substitutions per nucleotide position.

Disk diffusion tests showed that the bacterium was multiresistant to antimicrobial drugs, including most of the β-lactams, aminoglycosides, fluoroquinolones, colistin, fosfomycin, and tigecyclin. It remained susceptible to amoxicillin-clavulanate, ticarcillin-clavulanate, carbapenems, and trimethoprim-sulfamethoxazole. Interpretation of the susceptibility test was impossible before 48 hours incubation, i.e., 4 days after the first positive blood culture.

We describe in this report a case of human bacteremia due to *C. terrae*. This environmental organism behaving as an opportunistic pathogen was able to produce infection in a severely immunosuppressed woman. The source of bacteremia was not clearly established. Catheter-related bacteremia was not confirmed by culture of the CVC tip sent to the laboratory on September 3. Virulence factors contributing to the pathogenicity of *C. terrae* have not yet been well defined. The infection could have been favored by the immunosuppressive therapy, the profound leukopenia, and the extensive cutaneous detachment subsequently associated with methotrexate overdose in this patient. Unlike most susceptible environmental organisms, this bacterium probably was assisted by its multiresistance to antimicrobial drugs in producing infection. The intrinsic or acquired resistance of *C. terrae* to antimicrobial drugs has not yet been fully elucidated. The lack of commercially available biochemical gallery databases makes correct identification of this environmental organism difficult. It also underlines the usefulness of 16S rDNA sequencing for identification of unusual gram-negative bacilli isolated from immunocompromised hosts (*7*).
